# The *SLC6A3* gene possibly affects susceptibility to late-onset alcohol dependence but not specific personality traits in a Han Chinese population

**DOI:** 10.1371/journal.pone.0171170

**Published:** 2017-02-09

**Authors:** Chang-Chih Huang, Shin-Chang Kuo, Yi-Wei Yeh, Chun-Yen Chen, Che-Hung Yen, Chih-Sung Liang, Pei-Shen Ho, Ru-Band Lu, San-Yuan Huang

**Affiliations:** 1 Graduate Institute of Medical Sciences, National Defense Medical Center, Taipei, Taiwan, R.O.C; 2 Department of Psychiatry, Taipei Buddhist Tzu Chi General Hospital, New Taipei City, R.O.C; 3 Department of Psychiatry, Tri-Service General Hospital, Taipei, R.O.C; 4 Department Division of Neurology, Department of Internal Medicine, Chiayi Yang-Ming Hospital, Chiayi, Taiwan, R.O.C; 5 Department of Psychiatry, Beitou Branch, Tri-Service General Hospital, Taipei, Taiwan, R.O.C; 6 Department of Psychiatry, Institute of Behavior Medicine, College of Medicine, National Cheng Kung University, Tainan, R.O.C; Harvard Medical School, UNITED STATES

## Abstract

Dopaminergic dysfunction has an important role in the pathoetiology of alcohol dependence (AD). The purpose of this study was to determine whether the solute carrier family 6 member 3 (*SLC6A3*) gene (also known as the dopamine transporter *DAT* gene) was associated with AD, and whether variants in the *SLC6A3* locus were associated with specific personality traits in patients with AD. Sixteen polymorphisms in *SLC6A3* were analyzed using 637 patients with AD and 523 healthy controls. To reduce clinical heterogeneity, patients were classified into two subgroups: early-onset AD (EOAD) and late-onset AD (LOAD). The Tridimensional Personality Questionnaire was used to assess the personality traits novelty seeking (NS) and harm avoidance (HA) in the patients with AD. Using allele frequency and genotype distribution comparisons and logistic regression analysis, we found evidence of association between rs6350 and AD (*P* < 0.05). Following subgroup analysis, we confirmed evidence of an association in patients with LOAD (*P* = 0.003), but not in patients with EOAD. Heterozygous carriers of the A allele have a nearly 3 times greater risk to develop LOAD compared to individuals who do not have an A allele. Although we found that patients with AD had higher NS and HA scores compared to controls (*P* < 0.001), we did not find evidence of association between *SLC6A3* polymorphisms and either NS or HA in patients with AD using linear regression analysis. The findings from our study indicate that the *SLC6A3* gene may have a role in susceptibility to late-onset AD in the Han Chinese population.

## Introduction

Alcohol dependence (AD) is a complex disorder presenting a broad variability of clinical manifestations that involve multifactorial etiologies including genetic, behavioral, environmental, and personality factors [1]. The heritability of AD is estimated to be moderate to high ranging from 51% to 64% [2, 3]. In attempts to identify the genetic influence on AD susceptibility, genes involved in the dopaminergic system have drawn much attention during the recent decades. Evidence suggests that alcohol administration alters dopamine release [4–7]. In medicating dopaminergic activity, the dopamine transporter plays a central role in the human brain. Alcohol may directly affect the function of the dopamine transporter, although with inconsistent results. For example, in vitro studies found that alcohol increased dopamine release via inhibition of the dopamine transporter [8, 9], whereas a subsequent animal study reported an increase in efficiency of the dopamine transporter caused by alcohol administration [10]. These differences may be accounted for by site-specific dopamine transporter responses towards alcohol in different brain regions [11]. Therefore, dysfunction or altered function of the dopamine transporter may contribute towards the pathophysiology of AD.

The solute carrier family 6 member 3 (*SLC6A3*) gene (also known as the dopamine transporter *DAT* gene) consists of 15 exons and is found on chromosome 5p15.3. Polymorphisms at the *SLC6A3* locus have been implicated in gene expression, transcriptional activity, and protein availability [12, 13]. Because of conflicting findings regarding an association between *SLC6A3* and substance dependence [14, 15], the role of the *SLC6A3* gene in the pathogenesis of AD remains unclear. Evidence of an association between *SLC6A3* and AD was limited using few genetic markers. Meta-analyses that explored whether one of the most frequently tested *SLC6A3* polymorphisms, a 40-bp variable number tandem repeat (VNTR) in the 3′ untranslated region, was associated with AD have shown inconsistent results [16, 17]. In contrast, other studies found that several single nucleotide polymorphisms (SNPs) located in exons of the *SLC6A3* gene may functionally contribute to AD. For example, the A allele of rs27072 located in exon 15 was identified as a risk allele for AD in a Japanese population [18]. Furthermore, Lind et al. [15] found that the A allele of rs6350 located in exon 2 had a protective effect against harmful drinking behavior in a male Finnish population. However, because of the inconsistent findings supporting a role of the *SLC6A3* gene in AD, further study using a larger sample size and a different ethnic population is warranted.

Different subtypes of AD may have different underlying neurobiological etiologies [19]; furthermore, genetic contributions affect a narrowly defined phenotype of affected individuals (a subgroup) instead of the entire range of phenotypes that form the clinical characteristics of complex brain disorders [20]. A major factor for addiction may be the age of onset. Previous research has demonstrated that age of onset in patients with AD is associated with different genetic variants and activity of the serotonin transporter [21], response to antidepressants [22], and clinical characteristics [23]. Schmid and colleagues found that variants in *SLC6A3* were associated with increased alcohol consumption by individuals that started drinking early in life and proposed that gene-environment interactions may contribute to the development of AD [24]. Compared to patients with AD characterized by an early onset of AD (EOAD), patients with late onset AD (LOAD) have a prolonged interval from regular drinking to the development of AD, and they may be influenced largely by environmental factors [25]. Furthermore, patients with LOAD show reduced alcohol-drinking behavior with sertraline treatment [22], a finding that indicates a relationship between alcohol drinking behavior and depression in this subgroup of patients. Based on these findings, we posit that further investigation to determine whether *SLC6A3* variants are associated with AD in subgroups defined by the age of onset is warranted.

Personality factors may be major predictors for either the course or the outcome of AD [26, 27]. Using the model of Cloninger’s biosocial theory of personality, patients with AD have higher novelty seeking (NS) and harm avoidance (HA) scores than that found in controls [28]. Studies have found an association between the *SLC6A3* gene and personality traits defined by the Cloninger’s model in healthy individuals [29, 30] and patients with depression [31]. However, evidence demonstrating a relationship between the *SLC6A3* gene and personality traits of Cloninger’s model in patients with AD remains lacking. Therefore, the present study evaluated personality traits determined by the Tridimensional Personality Questionnaire (TPQ) in patients with AD to determine whether there was evidence of genetic contribution to personality.

In the present study, we performed a comprehensive association study using 16 genetic markers from the *SLC6A3* gene in a large sample of a Han Chinese population to investigate whether there was evidence of an association between *SLC6A3* variants with age of onset and specific personality traits in patients with AD.

## Materials and methods

### Subjects

The study protocol was approved by the Ethics Committee of Tri-Service General Hospital (TSGH) for the Protection of Human Subjects (TSGHIRB 096-05-073-A). All of the patients were recruited from the outpatient or inpatient departments of TSGH in Taipei, Taiwan. Following a detailed description of the study procedure, written informed consent was obtained from all participants. All of the study participants were recruited from July 1, 1997 to December 31, 2015 and they were unrelated to each other and ethnically Han Chinese.

The patient group consisted of 637 AD patients, of whom 229 are classified with EOAD (age < 24 y/o) and 408 with LOAD (age ≥ 25 y/o). Each patient was diagnosed AD based on the Diagnostic and Statistical Manual of Mental Disorders, Fourth Edition (DSM-IV-TR) by a general psychiatrist initially in clinical inpatient or outpatient settings and subsequently interviewed by either a well-trained psychologist or a psychiatrist using a Chinese Version of the Modified Schedule of Affective Disorder and Schizophrenia-Life Time (SADS-L) [32]. Patients with a history of other substance dependence (except nicotine), organic brain disease, severe medical illness, or any concomitant major psychiatric disorders were excluded from this study.

The control group comprised 436 healthy volunteers from the community. We used SADS-L to exclude past or present psychiatric illnesses including affective disorders, schizophrenia, anxiety disorders, personality disorders, and substance use disorders in this group. Moreover, subjects with positive family history of psychiatric disorders presented in first-degree relatives were excluded.

### Selection of genetic variants and genotyping methods of the *SLC6A3* gene

Genomic DNA from the participants was extracted from peripheral blood leukocytes by a commercial kit (DNAzol; Invitrogen, Carlsbad, CA, USA). The study variants were selected according to the human *SLC6A3* polymorphisms in the HapMap database (www.hapmap.org), NCBI SNP database (www.ncbi.nlm.nih.gov/projects/SNP/), and a relevant review of literature [15, 33]. A total of 16 SNP polymorphisms were investigated to cover a region of 56 kb in the *SLC6A3* gene: three SNPs [rs2550948, rs2652511, and rs2975226] in the promoter region, one [rs6350] in exon 2, one [rs2981359] in intron 2, two [rs403636, rs460000] in intron 3, two [rs460700, rs464049] in intron 4, one [rs37020] in intron 6, one [rs37022] in intron 7, one [rs27048] in intron 8, one [rs6347] in exon 9, one [rs11133767] in intron 13, one [rs40184] in intron 14, and one [rs27072] in exon 15. All SNPs of the *SLC6A3* gene were genotyped by TaqMan assays (Applied Biosystems, Foster City, CA) with Applied Biosystems StepOne^™^ software and StepOnePlus^™^ Real-Time PCR System (Applied Biosystems, Foster City, CA).

### Assessment of personality traits

The Chinese version of the Tridimensional Personality Questionnaire (TPQ) was used to evaluate personality traits in patient and control groups [34]. We analyzed only NS (32 items, Cronbach's α = 0.70) and HA (34 items, Cronbach's α = 0.87) dimensions but not reward-dependence dimension (RD, 34 items, Cronbach's α = 0.54) because of low inter-rater reliability of RD among Han Chinese in Taiwan [34]. 198 AD patients and 252 controls who completed the TPQ were eligible for subsequent analysis. Subjects in AD group were assessed after a withdrawal period in order to avoid confounding effects as a result of alcohol withdrawal symptoms.

### Statistical analysis

We employed an independent sample *t* test and two-tailed Pearson test to analyze the differences in mean age, gender, allele and genotype frequencies for each SNPs between the AD patients and controls. Fisher’s exact test was substituted for chi-square test when the sample size was smaller than expected. Multivariate logistic regression analysis was used to correct the effect of possible covariates such as age, gender, and gene-to-gene interactions in the risk for AD. For personality trait assessment, a linear regression analysis was conducted using each SNP as an independent variable, with patient/control group, gender, and age as covariates, and NS or HA score as dependent variables. All statistical analyses were performed using SPSS statistical software (version 19, SPSS Inc., Chicago, IL, USA).

For haplotype association analysis, we analyzed the linkage disequilibrium (LD) coefficients (D’), haplotype frequency, haplotype block, haplotype association, and Hardy-Weinberg equilibrium for each variant using Haploview V4.1 software (Broad Institute, Cambridge, MA) [35], in which we defined a haplotype block as a set of contiguous SNPs with an average D’ greater than 0.9. All tests were two-tailed, and alpha was set at 0.05. To correct the halplotype analysis for multiple testing, 10,000 permutations were performed for haplotype-specific *P*-values.

## Results

### Demographic data

Of the 1,185 participants enrolled in this study, 25 were excluded because of incomplete genotype data. The final 1,160 subjects that were used in our analysis included 637 patients with AD and 523 healthy controls. The mean age of onset for AD group was 30.62 ± 9.52 years. There were no differences in age between the two groups (mean age of 39.36 ± 11.23 years for patients vs. mean age of 39.84 ± 12.32 years for controls, *P* = 0.490); however, our AD cohort had a higher proportion of males than females compared to the control group (88.2% vs. 69.2% respectively, *P* < 0.001). Furthermore, 256 patients (40.19%) with AD were recruited from inpatient department and there was no difference in gender and age compared to those recruited from outpatient department (*P* > 0.05).

### *SLC6A3* variants in patients with AD

Genotype distributions and allele frequencies in AD and control cohorts are summarized in Table 1. All variants were in Hardy-Weinberg equilibrium in the control group. We found an increased frequency of the rs6350 A allele and of the A/G genotype in patients with AD compared to that found in controls (uncorrected *P* = 0.008 and *P* = 0.008, respectively). We found in our subgroup analysis of genotype distribution comparisons that this association was with the LOAD group (uncorrected *P* = 0.003), but not with the EOAD group (*P* = 0.346, as shown in S1 Table). Following conservative adjustments for multiple comparisons, our finding in the LOAD subgroup remained significant (adjusted *P* = 3.13 × 10^−3^). To account for potentially confounding effects because of differences in gender distribution between patient and control cohorts, we performed multivariate logistic regression analysis in our efforts to identify putative risk alleles from 16 SNPs in the *SLC6A3* gene associated with AD and its subgroups (Table 2). We confirmed that rs6350 in the *SLC6A3* gene was associated with the pathogenesis of AD (*P* = 0.012), and the association became more significant in the LOAD subgroup (*P* = 0.005); heterozygous carriers of the A allele have a nearly 3 times greater risk to develop LOAD compared to individuals who do not have an A allele.

**Table 1:** Genotype distributions and allelic frequencies of the polymorphisms in the *SLC6A3 (DAT)* gene between patients with alcohol dependence and controls in a Han Chinese population.

Variant	Position reference dSNP	Allele ^a^		MAF ^a^ (%)		*p* ^b^	Genotype (%)										*p* ^b^
		1	2	Control	AD		Control (N = 523)			AD (N = 637)			*p* ^b^	LOAD (N = 408)			
							1/1	1/2	2/2	1/1	1/2	2/2		1/1	1/2	2/2	
**rs2550948**	**1503444(P)**	***A***	***G***	13.00	15.78	0.059	10 (1.9)	116 (22.2)	397 (75.9)	12 (1.9)	177 (27.8)	448 (70.3)	0.091	9 (2.2)	114 (27.9)	285 (69.9)	0.115
**rs2652511**	**1499389(P)**	***C***	***T***	14.34	16.56	0.142	12 (2.3)	126 (24.1)	385 (73.6)	12 (1.9)	187 (29.4)	438 (68.8)	0.126	9 (2.2)	121 (29.7)	278 (68.1)	0.161
**rs2975226**	**1498616(P)**	***A***	***T***	14.63	16.56	0.202	13 (2.5)	127 (24.3)	383 (73.2)	12 (1.9)	187 (29.4)	438 (68.8)	0.134	9 (2.2)	121 (29.7)	278 (68.1)	0.183
**rs6350**	**1496199(E2)**	***A***	***G***	1.24	2.83	0.008	0 (0)	13 (2.5)	510 (97.5)	0 (0.0)	36 (5.7)	602 (94.5)	0.008^c^	0 (0)	27 (6.6)	381 (93.4)	0.003^c^
**rs2981359**	**1495732(In2)**	***G***	***C***	43.31	44.03	0.726	95 (18.2)	263 (50.3)	165 (31.5)	124 (19.5)	313 (49.1)	200 (31.4)	0.845	81 (19.9)	202 (49.5)	125 (30.6)	0.805
**rs403636**	**1491354(In3)**	***A***	***C***	32.98	33.20	0.911	53 (10.1)	239 (45.7)	231 (44.2)	73 (11.5)	277 (43.5)	287 (45.1)	0.660	41 (10.0)	183 (44.9)	184 (45.1)	0.960
**rs460000**	**1485825(In3)**	***G***	***T***	46.08	48.51	0.244	104 (19.9)	274 (52.4)	145 (27.7)	147 (23.1)	324 (50.9)	166 (26.1)	0.411	87 (21.3)	221 (54.2)	100 (24.5)	0.531
**rs460700**	**1482969(In4)**	***T***	***C***	47.04	49.84	0.178	111 (21.2)	270 (51.6)	142 (27.2)	157 (24.6)	321 (50.4)	159 (25.0)	0.355	97 (23.8)	216 (52.9)	95 (23.3)	0.352
**rs464049**	**1476905(In4)**	***A***	***G***	36.42	36.50	0.970	69 (13.2)	243 (46.5)	211 (40.3)	94 (14.8)	277 (43.5)	266 (41.8)	0.547	54 (13.2)	184 (45.1)	170 (41.7)	0.909
**rs37020**	**1471374(In6)**	***A***	***C***	35.37	36.34	0.628	61 (11.7)	248 (47.4)	214 (40.9)	93 (14.6)	277 (43.5)	267 (41.9)	0.233	59 (14.5)	178 (43.6)	171 (41.9)	0.339
**rs37022**	**1468629(In7)**	***T***	***A***	50.38	47.33	0.144	137 (26.2)	253 (48.4)	133 (25.4)	153 (24.0)	297 (46.6)	187 (29.4)	0.311	101 (24.8)	182 (44.6)	125 (30.6)	0.210
**rs27048**	**1465645(In8)**	***T***	***C***	15.68	15.86	0.908	12 (2.3)	140 (26.8)	371 (70.9)	9 (1.4)	184 (28.9)	444 (69.7)	0.416	6 (1.5)	116 (28.4)	286 (70.1)	0.589
**rs6347**	**1464412(E9)**	***G***	***A***	12.43	11.15	0.339	4 (0.8)	122 (23.3)	397 (75.9)	10 (1.6)	122 (19.2)	505 (79.3)	0.111^c^	5 (1.2)	84 (20.6)	319 (78.2)	0.518^c^
**rs11133767**	**1454580(In13)**	***T***	***C***	11.19	10.13	0.409	14 (2.7)	89 (17.0)	420 (80.3)	11 (1.7)	107 (16.8)	519 (81.5)	0.533	7 (1.7)	72 (17.6)	329 (80.6)	0.608
**rs40184**	**1448077(In14)**	***T***	***C***	25.53	25.12	0.822	30 (5.7)	207 (39.6)	286 (54.7)	37 (5.8)	246 (38.6)	354 (55.6)	0.946	23 (5.6)	147 (36.0)	238 (58.3)	0.521
**rs27072**	**1447522(E15)**	***T***	***C***	27.44	24.65	0.127	45 (8.6)	197 (37.7)	281 (53.7)	37 (5.8)	240 (37.7)	360 (56.5)	0.167	26 (6.4)	152 (37.3)	230 (56.4)	0.407

MAF, minor allele frequency; P, promoter; E, exon; In, intron; AD: Alcohol dependence; LOAD: late-onset alcohol dependence.

^a^ Allele 1 is the minor allele, and only alleles with frequency higher than 1% are shown.

^b^ Indicated genotype or allelic frequencies in patients with AD or LOAD compared with the control group.

^c^ Statistical analysis was performed by Fisher’s exact test.

**Table 2:** Multivariate logistic regression analyses of the sixteen polymorphisms of *SLC6A3 (DAT)* gene as risk factors for alcohol dependence (AD) and its subgroups in a Han Chinese population.

	AD (N = 637)		LOAD (N = 408)		EOAD (N = 229)	
Variants^a^	Odds ratio (95% CI)	*p* value^c^	Odds ratio (95% CI)	*P* value^c^	Odds ratio (95% CI)	*p* value^c^
***rs2550948*** *(G/G)*						
*A/G and A/A* ^b^	1.82 (0.88–3.78)	0.108	1.98 (0.86–4.55)	0.107	1.87 (0.58–6.08)	0.297
***rs2652511*** *(T/T)*						
*C/T and C/C*	1.71 (0.26–11.44)	0.580	1.19 (0.16–8.73)	0.863	2.41 (0.10–58.78)	0.589
***rs2975226*** *(T/T)*						
*A/T and A/A* ^b^	0.34 (0.05–2.35)	0.276	0.46 (0.06–3.40)	0.450	0.24 (0.01–6.68)	0.403
***rs6350*** *(G/G)*						
*G/A*	2.52 (1.23–5.17)	0.012	2.99 (1.40–6.40)	0.005	1.86 (0.64–5.35)	0.253
***rs2981359*** *(C/C)*						
*G/C*	0.96 (0.73–1.28)	0.797	0.91 (0.66–1.26)	0.577	1.02 (0.68–1.54)	0.921
*G/G*	0.88 (0.61–1.27)	0.501	0.87 (0.57–1.32)	0.502	0.85 (0.49–1.46)	0.550
***rs403636*** *(C/C)*						
*A/C*	0.84 (0.40–1.75)	0.635	0.66 (0.29–1.50)	0.318	1.08 (0.36–3.25)	0.888
*A/A*	0.80 (0.31–2.05)	0.642	0.73 (0.25–2.11)	0.561	0.97 (0.24–3.93)	0.964
***rs460000*** *(T/T)*						
*G/T*	0.89 (0.34–2.38)	0.819	0.48 (0.16–1.42)	0.184	6.83 (0.69–68.03)	0.101
*G/G*	0.64 (0.15–2.67)	0.542	0.38 (0.07–1.96)	0.246	2.66 (1.74–40.67)	0.481
***rs460700*** *(C/C)*						
*C/T*	1.25 (0.41–3.85)	0.698	1.64 (0.40–6.78)	0.495	1.14 (0.26–5.05)	0.865
*T/T*	1.58 (0.40–6.17)	0.512	2.96 (0.61–14.38)	0.178	0.31 (0.02–4.68)	0.395
***rs464049*** *(G/G)*						
*G/A*	0.73 (0.33–1.61)	0.438	0.53 (0.21–1.31)	0.167	1.21 (0.43–3.38)	0.714
*A/A*	0.70 (0.24–1.98)	0.495	0.46 (0.13–1.55)	0.207	1.64 (0.37–7.18)	0.514
***rs37020*** *(C/C)*						
*A/C*	0.95 (0.47–1.92)	0.888	0.87 (0.33–2.78)	0.729	0.83 (0.30–2.24)	0.702
*A/A*	1.71 (0.65–4.54)	0.279	2.57 (0.84–7.86)	0.098	0.68 (0.17–2.72)	0.580
***rs37022*** *(A/A)*						
*T/A*	0.87 (0.59–1.30)	0.502	0.68 (0.43–1.06)	0.085	1.52 (0.82–2.80)	0.184
*T/T*	0.74 (0.43–1.28)	0.287	0.77 (0.41–1.46)	0.425	0.69 (0.30–1.55)	0.365
***rs27048*** *(C/C)*						
*C/T and T/T* ^b^	1.16 (0.82–1.64)	0.407	1.19 (0.80–1.77)	0.400	1.15 (0.70–1.91)	0.582
***rs6347*** *(A/A)*						
*A/G and G/G* ^b^	0.74 (0.52–1.07)	0.112	0.83 (0.55–1.26)	0.381	0.59 (0.34–1.01)	0.056
***rs11133767*** *(C/C)*						
*C/T and T/T* ^b^	1.13 (0.76–1.68)	0.547	1.34 (0.85–2.11)	0.210	0.85 (0.48–1.51)	0.588
***rs40184*** *(C/C)*						
*C/T and T/T* ^b^	0.98 (0.73–1.31)	0.867	0.73 (0.52–1.04)	0.078	1.48 (0.97–2.27)	0.069
***rs27072*** *(C/C)*						
*C/T and T/T* ^b^	0.78 (0.58–1.04)	0.087	0.79 (0.57–1.11)	0.172	0.68 (0.44–1.03)	0.069

AD: Alcohol dependence; LOAD: late-onset alcohol dependence; EOAD: early-onset alcohol dependence.

Age and gender are used as covariates.

^a^ Genotype within parenthesis indicates the reference group of genotype.

^b^ The minor genotype frequencies are combined if the frequency is less than 10%.

^c^ Odds ratio is given with 95% CI after using a logistic regression analysis compared with controls.

### Haplotype analysis in the *SLC6A3* gene

**Fig 1:**
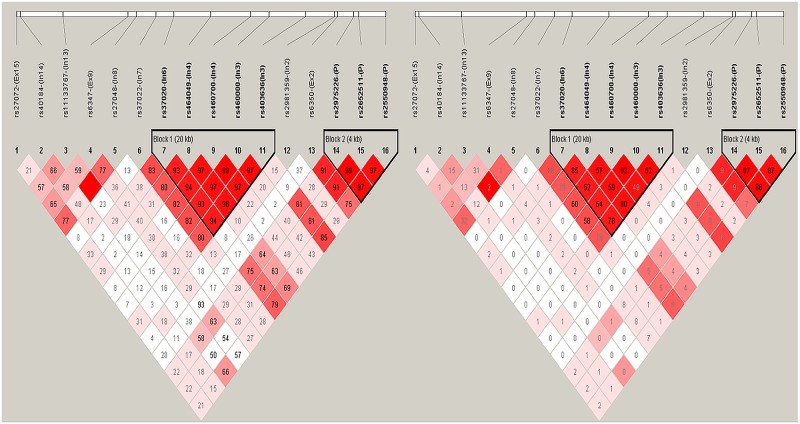
The linkage disequilibrium (LD) structure between 16 SNP polymorphisms in *SLC6A3* gene. The upper panel shows the location of 16 polymorphisms in *SLC6A3* gene and lower panel shows the output of Haploview version 4.1. D’ (left) and r^2^ value (right) were shown within each square represents a pairwise linkage disequilibrium relationship between two polymorphisms. Red squares indicate statistically significant LD between the pair of polymorphisms. Darker colors of red indicate higher values of D’ or r^2^, up to a maximum of 1 and white squares indicate pairwise D’ values with no statistically significant difference of LD. The two haplotype blocks generated under confidence interval algorithm of haploview are marked. The definitions of the abbreviations: Ex, exon; In, intron; P, promoter.

SNP locations at the *SLC6A3* gene, linkage disequilibrium (LD) structure, and D′ values for all variants used in this study are shown in Fig 1. A haplotype block was defined as a set of contiguous SNPs with an average D′ greater than 0.9. We identified two major haplotype blocks using these 16 polymorphisms at the *SLCC6A3* locus in our cohorts (Table 3). Block 1 is 20 kb in size and includes five SNPs (rs37020, rs464049, rs460700, rs460000, and rs403636) spanning the region between intron 3 and intron 6, while block 2 is 4.8 kb in size and includes three SNPs (rs2975226, rs2652511, and rs2550948) spanning the promoter region. Among all subjects, no significant difference was observed for these two blocks between patients with AD and controls.

**Table 3:** Haplotype analysis of *SLC6A3 (DAT)* gene in patients with alcohol dependence (N = 637) and controls (N = 523). (Haplotype frequencies>0.01).

**Haplotype block 1:**					**Frequency**		***p***
***rs37020***	***rs464049***	***rs460700***	***rs460000***	***rs403636***	**AD**	**Control**	
**C**	**G**	**C**	**T**	**C**	0.487	0.512	0.236
**A**	**A**	**T**	**G**	**A**	0.318	0.311	0.711
**C**	**G**	**T**	**G**	**C**	0.117	0.100	0.209
**A**	**A**	**T**	**G**	**C**	0.029	0.026	0.648
**C**	**G**	**C**	**T**	**C**	0.014	0.007	0.085
**Haplotype block 2:**					**Frequency**		***p***
***rs2975226***		***rs2652511***		***rs2550948***	**AD**	**Control**	
**T**		**T**		**G**	0.829	0.851	0.159
**A**		**C**		**A**	0.153	0.187	0.094
**A**		**C**		**G**	0.013	0.013	0.855

### Relationship between *SLC6A3* variants and personality scores

One hundred and ninety-eight patients with AD and two hundred and fifty-two controls completed the TPQ and were available for gene-personality analysis. The scores for both HA and NS were higher in the patient cohort than that found in the control cohort (17.24 ± 4.91 vs. 12.61 ± 4.63 respectively, *P* < 0.001 for NS; 16.63 ± 6.22 vs. 10.58 ± 5.28 respectively, *P* < 0.001 for HA). No difference were observed in the HA scores between patients from the EOAD (N = 68) and LOAD (N = 130) subgroups who completed the TPQ (16.65 ± 5.32 for EOAD vs. 16.62 ± 6.67 for LOAD, *P* = 0.978). However, patients with EOAD had higher NS scores than the patients with LOAD (18.60 ± 4.76 vs. 16.53 ± 4.85 respectively, *P* = 0.004). Using one-way ANOVA to analyze all three groups together (EOAD, LOAD, and controls), we found a decreasing NS score in the order EOAD > LOAD > controls (*P* < 0.001).

To exclude potential effects from age of onset and gender bias, we performed linear regression analysis to investigate further whether *SLC6A3* variants were associated with specific personality traits in patients with AD (Table 4). Of the 16 *SLC6A3* polymorphisms tested, rs464049 and rs37020 were associated with NS (*P* = 0.024 and *P* = 0.008, respectively), and several SNPs were associated with HA (*P* = 0.013 for rs2550948, *P* = 0.016 for rs2652511, *P* = 0.016 for rs2975226, and *P* = 0.036 for rs403636). However, none of these associations remained following Bonferroni corrections for multiple comparisons. Furthermore, gender was not associated with either NS or HA scores; in contrast, we found that age of onset was negatively correlated with NS in patients with AD (*P* < 0.001).

**Table 4:** Linear regression analysis between *SLC6A3 (DAT)* gene polymorphisms and personality traits in patients with AD (215 patients completed TPQ).

Variants	Novelty Seeking ^a^					Harm Avoidance ^a^				
	Genotype	Age	Gender	Age of onset		Genotype	Age	Gender	Age of onset	
	β (SE)	β (SE)	β (SE)	β (SE)	*p* value ^b^	β (SE)	β (SE)	β (SE)	β (SE)	*p* value ^b^
rs2550948	−0.625 (0.613)	−0.184 (0.033)	0.287 (1.015)	−0.132 (0.766)	0.309	2.059 (0.825)	−0.116 (0.044)	−2.012 (1.366)	1.396 (1.032)	0.013
rs2652511	−0.679 (0.612)	−0.185 (0.033)	0.301 (1.015)	−0.147 (0.767)	0.268	2.004 (0.825)	−0.116 (0.044)	−2.011 (1.368)	1.417 (1.034)	0.016
rs2975226	−0.679 (0.612)	−0.185 (0.033)	0.301 (1.015)	−0.147 (0.767)	0.268	2.004 (0.825)	−0.116 (0.044)	−2.011 (1.368)	1.417 (1.034)	0.016
rs6350	−0.007 (1.473)	−0.184 (0.033)	0.167 (1.019)	−0.070 (0.766)	0.996	1.133 (2.008)	−0.118 (0.045)	−1.517 (1.389)	1.182 (1.044)	0.573
rs2981359	0.031 (0.431)	−0.184 (0.033)	0.163 (1.013)	−0.067 (0.767)	0.931	−0.297 (0.588)	−0.117 (0.045)	−1.575 (1.381)	1.160 (1.046)	0.613
rs403636	1.048 (0.534)	−0.186 (0.033)	0.387 (1.007)	−0.001 (0.759)	0.051	−1.534 (0.727)	−0.115 (0.044)	−1.939 (1.372)	1.090 (1.034)	0.036
rs460000	0.584 (0.467)	−0.185 (0.033)	0.133 (1.007)	−0.002 (0.765)	0.212	−0.195 (0.639)	−0.118 (0.045)	−1.606 (1.379)	1.167 (1.047)	0.760
rs460700	−0.646 (0.463)	−0.185 (0.033)	0.103 (1.007)	−0.019 (0.763)	0.165	0.113 (0.635)	−0.118 (0.045)	−1.607 (1.380)	1.181 (1.046)	0.858
rs464049	1.115 (0.491)	−0.186 (0.032)	0.324 (1.000)	−0.003 (0.757)	0.024	−1.349 (0.671)	−0.115 (0.044)	−1.807 (1.368)	1.109 (1.035)	0.046
rs37020	−1.302 (0.485)	−0.183 (0.032)	0.366 (0.995)	−0.123 (0.752)	0.008	1.232 (0.668)	−0.119 (0.044)	−1.806 (1.371)	1.240 (1.036)	0.067
rs37022	0.474 (0.430)	−0.184 (0.033)	0.130 (1.008)	−0.053 (0.764)	0.272	−0.176 (0.588)	−0.118 (0.045)	−1.604 (1.379)	1.184 (1.045)	0.765
rs27048	0.757 (0.620)	−0.179 (0.033)	0.187 (1.007)	−0.124 (0.764)	0.223	−0.982 (0.846)	−0.125 (0.045)	−1.643 (1.374)	1.261 (1.043)	0.247
rs6347	0.126 (0.763)	−0.184 (0.033)	0.175 (1.012)	−0.068 (0.766)	0.869	−0.601 (1.040)	−0.118 (0.045)	−1.652 (1.379)	1.181 (1.044)	0.564
rs11133767	−0.199 (0.744)	−0.184 (0.033)	0.182 (1.012)	−0.064 (0.766)	0.790	1.159 (1.011)	−0.118 (0.045)	−1.703 (1.376)	1.155 (1.011)	0.253
rs40184	0.076 (0.552)	−0.183 (0.034)	0.170 (1.011)	−0.093 (0.785)	0.891	−0.932 (0.750)	−0.131 (0.046)	−1.655 (1.374)	1.481 (1.067)	0.215
rs27072	0.057 (0.523)	−0.184 (0.033)	0.167 (1.011)	−0.064 (0.768)	0.914	1.336 (0.706)	−0.127 (0.045)	−1.628 (1.366)	1.321 (1.037)	0.055

AD, alcohol dependence; TPQ, Tridimensional Personality Questionnaire.

^a^ The association between all *SLC6A3* variants and TPQ score are corrected for age, gender, and age of onset as covariates using linear regression analysis.

^b^ The *p* value for the relationship between TPQ score and each *SLC6A3* variant.

## Discussion

Although the role of the dopamine transporter in dopaminergic neurotransmission makes the *SLC6A3* gene an appealing candidate gene for AD, evidence of an association and identification of susceptibility alleles to AD has been controversial and non-reproducible [16, 36]. To our knowledge, this is the first case-control study using single marker and haplotype analyses with multiple SNPs to determine whether there is evidence of a role of the *SLC6A3* gene in patients with AD in a Han Chinese population. Using allele frequency and genotype distribution comparisons, and logistic regression analysis, we found evidence of an association with the A allele of the exonic SNP rs6350 in patients with AD compared to that found in controls. However, not all findings remained statistically significant following corrections for multiple comparisons. In fact, our major finding was in patients from the LOAD subgroup, but not the EOAD subgroup. In the present study, the minor A allele of rs6350 was considered a risk allele for LOAD and that heterozygous carriers have a nearly 3 times increased risk to develop LOAD compared to individuals who do not have the A allele. Our findings are in contrast to the findings of Lind et al. [15] in which the rs6350 A allele was a protective allele associated with decreased severity of problem drinking and incidence of AD. Several factors may account for these observed differences between our studies. First, there can be ethnic- and population-specific differences in allele frequencies and genotype distributions of genetic polymorphisms. The minor allele frequency of rs6350 was approximately 2.2% in our Han Chinese controls, a frequency that is considerably lower than the 10.5% found in controls from a Finnish population [15]. To decrease the risk of bias because of sample selection and to increase the statistical power of our study, we used a large sample size recruiting 1,160 individuals. In addition, we recruited both males and females with AD instead of specifically testing only affected males in our investigation of the role of the *SLC6A3* gene in AD. We also used restricted inclusion criteria for our control subjects, excluding individuals if they met the diagnosis of AD and showed alcohol abuse as determined using the SADS-L. However, individuals recruited in the control cohort of the study by Lind et al. were assessed solely by the Alcohol Use Disorders Identification Test (AUDIT) and furthermore, they were categorized as either pure controls or problem drinkers. This heterogeneity in exclusion and inclusion criteria between the control cohorts of our two studies may also contribute to the differences in their respective findings. Finally, the contradictory AD allele of rs6350 in the two populations might be derived from high recombination between this SNP and a disease locus. However, both Lind et al. and we conclude that rs6350 at the *SLC6A3* gene has an association with AD. Whether this synonymous exonic SNP is in linkage disequilibrium with other *SLC6A3* variants that could alter transporter availability or function in the human brain warrants further investigation.

Genetic polymorphisms at the 5′ UTR promoter region of *SLC6A3* have been proposed to be functional and known to alter transcriptional activity [12], and that these variants found in the promoter region may have a role in several psychiatric disorders, including attention deficit-hyperactivity disorder [37], bipolar disorder [38], and schizophrenia [39]. In the present study, we used three common SNPs to explore the significance of *SLC6A3* promoter for AD. However, neither single marker nor haplotype analyses revealed an association between patients with AD and controls. Therefore, despite the fact that *SLC6A3* promoter variants are excellent candidates for linkage and association studies, we did not find evidence supporting their role in AD in our cohort of Han Chinese population. For the most investigated 3' UTR VNTRs in relation to AD, two meta-analyses showed inconsistent results [16, 17]. The contradictory findings might be attributable to differences in study design such as data sampling and statistical methods. In the latter study, for example, Ma et al. [17] concluded that the 9-repeat allele increased the risk for AD in different populations using a dominant genetic model with a larger sample size. However, the authors did not include two studies that considered that the 9-repeat allele was protective for AD in Japanese and Han Chinese populations [40, 41]. Another frequently tested polymorphism located in the 3′ region of *SLC6A3* that is tested for association with psychiatric disorders is rs27072, which is in exon 15. Ueno et al. [18] investigated the role of rs27072 in AD in a Japanese population and found that the minor A allele was a risk allele in their AD cohort. In comparison, we found that the minor allele frequency (MAF) of rs27072 was comparable between our affected cohort (24.7%) and that of Ueno et al. (26.6%). However, they had a markedly lower MAF in their control group (17.3%) compared to that found in ours (27.4%). Therefore, it is likely that the MAF differences between the control groups accounts for the differences in findings between the two studies. Based on our findings, we conclude that polymorphisms from either the 5′ or 3′ region of the *SLC6A3* gene are not associated with AD in our Han Chinese population.

Another factor that may account for the observed heterogeneity between findings in genetic association studies is that interactions between different genetic and environmental factors modulate susceptibility to AD. Therefore, subgroup analysis using age of onset of AD has been used as a means to resolve conflicting findings and increase homogeneity in genetic association studies [21, 22]. In the present study, and following adjustments for multiple comparisons, the rs6350 polymorphism was identified to confer risk to develop AD, specifically in patients from the LOAD subgroup, but not in patients from the EOAD subgroup. In Cloninger's model of alcoholism, type I alcoholism is characterized by anxiety proneness, loss of control and excessive guilt over alcohol intake, over 25 years of age, and progressive severity of alcohol abuse. Individuals with type I alcoholism initially inhibit drinking behavior because of their proneness to anxiety and therefore, experience a later onset of alcoholism. However, they may be characterized as having a binge drinking pattern with a rapid progression, and accompanied by the development of alcoholic liver disease later in life [42]. It is proposed that individuals with type I alcoholism have a greater environmental predisposition than individuals with type II alcoholism. For example, adoptive studies found that both males and females were equally likely to develop type I alcoholism, and that they may have more severe and frequent alcohol abuse if their adoptive fathers have a low occupational status [42]. In addition, there may be neurobiological differences underlying type I and II alcoholism, owing to which individuals with type I alcoholism are more vulnerable to dopaminergic deficits, and individuals with type II alcoholism have deficits in serotonergic neurotransmission [43]. We hypothesize that patients in our LOAD subgroup have deficits in the dopaminergic system because of dysfunction or altered function of the dopamine transporter. Depression is another potential factor that may confound the role of *SLC6A3* in patients with LOAD. Previous studies found that depressive symptoms are risk factors for LOAD [44], and that patients with LOAD have significantly reduced alcohol drinking and favorable outcomes following treatment with antidepressants [45]. Although Kranzler et al. [22] posited a role for the serotonergic system towards susceptibility of AD among patients with LOAD, we propose that dopaminergic deficits contribute to both depression and addictive disorders, and are responsible for the comorbidity found between depression and AD. We hypothesize that individuals with the rs6350 A allele show dysfunction or altered dopaminergic function, and that this genetic predisposition increases vulnerability to environmental stressors or exacerbates a predisposition to depression in patients with LOAD. Consequently, we speculate that these patients adopted a self-medication pattern of alcohol drinking behavior for their sub-clinical depression, which occurs later in life and contributes to the late-onset of AD found in these patients. In the present study, we could not examine environmental factors such as life stressors or childhood negative experiences, or evaluate the presence or severity of depressive symptoms in our cohorts. Nevertheless, it is warranted that future studies are performed to investigate these factors.

Because pathologies that underlie comorbid personality traits may contribute or have an effect on the course or prognosis of AD, specific personality traits may reflect a genetic predisposition for substance abuse [26, 27]. Our patients with AD had higher NS and HA scores compared to the healthy controls, findings that are in line with those from an earlier study [28]. In our subgroup analysis, we found that patients with EOAD had the highest NS scores, followed by patients with LOAD, and controls. In contrast, no association was found between our subgroups and controls using HA scores. It was proposed that NS is a highly inherited personality trait [46], and similar to addiction, is modulated by the dopaminergic system [47]. Individuals with a high NS score are more likely to experiment with drugs at an early age, to be attracted to novel stimuli, and to respond rapidly to cues for reward despite potential punishment. Therefore, it was considered that a high NS score was a predictor for an increased risk of relapse, cravings, and the compulsive use of alcohol in patients with AD [48, 49]. However, evidence demonstrating that NS and addictive behaviors are affected by dysfunction of altered function of the dopaminergic system because of variants in *SLC6A3* is limited. For healthy controls, both association and non-association between NS and *SLC6A3* polymorphisms have been reported [30, 50, 51]. Therefore, researchers argued that the impact of genes on specific personality traits might be due to gene-gene interactions rather than a single genetic polymorphism. To minimize or avoid potential confounding effects caused by age of onset or gender, we used linear regression analysis instead of one-way ANOVA in our evaluation. Although evidence of associations between two *SLC6A3* intronic variants (rs464049 and rs37020) and NS was found, the findings were not significant following corrections for multiple testing. However, our finding that there is no association is comparable to previous studies that did not find evidence of association in patients with substance use disorders such as amphetamine dependence [52] and heroin dependence [53]. Anghelescu and colleagues proposed that the 3′ VNTR polymorphism of the *SLC6A3* gene may affect NS in Caucasian patients with AD. They found that a small subgroup of patients homozygous for the 10 allele had lower NS tendencies compared to that found in other patients with AD and controls [54]. Although we did not directly test the 3′ VNTR in our study, we used multiple genetic markers to comprehensively cover the *SLC6A3* gene. In addition, we performed strict statistical analyses incorporating adjustments for multiple comparisons, and used a large sample size to confirm that there was no evidence of an association between the *SLC6A3* gene and NS scores in our cohort of patients with AD. In line with the current understanding that HA is determined by the serotonergic system of the brain, we found no evidence of an association between our tested SNPs in *SLC6A3* and HA scores in our cohort of patients with AD. Therefore, although we found higher scores for NS and HA personality traits in patients with AD compared to that found in controls, we found no evidence of a role for the *SLC6A3* gene in influencing these specific personality traits in an AD cohort from the Han Chinese population.

We identified several limitations in the present study. First, despite enrolment of a relatively large number of participants, there may be insufficient power to robustly detect associations within patient subgroups and in particular, the EOAD subgroup. Our total sample size (N = 752) for EOAD subgroup analysis only had the power of 0.69 to detect a small effect. Second, we observed a disequilibrium in the sex ratio between our controls and that found in our AD cohort. To account for this observed gender bias, we analyzed our data only in male subjects (as shown in S2 Table). Although the results were similar to that obtained using total subjects, we only detected nominal association for rs6350 after multiple corrections between case and control groups, including that for LOAD subgroup. This finding might be attributable to the insufficient sample size for male subjects and warrants further investigation. Third, we used a dichotomized method with an age of onset at 25 years, rather than a detailed age distribution, for the subgroup analysis, which may not be sufficient to apply in real situation. Fourth, although personality scores were not determined while patients were intoxicated or in a state of withdrawal, the possible confounding effects of medications such as soporifics or sedatives on personality traits cannot be ruled out. In addition, we used Cloninger’s biosocial theory that considers NS and HA, but not other personality traits in the Temperament and Character Inventory such as persistence, self-directedness, cooperativeness, and self-transcendence. Therefore, the lack of evidence supporting genetic contribution to personality in our study does not represent the entire personality dimension, but is specific to that of NS and HA in our patient cohort. Moreover, ethnic or population differences in allele or genotype frequencies of genetic variants or environmental factors may have important roles in the pathogenesis of AD, particularly, of LOAD. However, the influence of environmental factors on the disease was not examined in the present study. Finally, the MAF of rs6350 was very low (less than 3%) and we did not find any individuals homozygous for the A allele in our study. Further investigation to determine whether this exonic *SLC6A3* gene variant has a role in the development of AD in individuals later in life is warranted.

## Conclusion

Using single marker and haplotype analyses, we found evidence of an association between the *SLC6A3* gene and susceptibility to AD. Following adjustments for multiple comparisons, evidence of this association remained in patients from the LOAD subgroup, but not the EOAD subgroup in our study of a Han Chinese population. In addition, we found no evidence for a role of the *SLC6A3* gene in personality traits in our cohort of patients with AD. Further gene-environment association studies are warranted to confirm our findings.

## Supporting information

S1 Fig The linkage disequilibrium (LD) structure between 16 SNP polymorphisms in *SLC6A3* gene for patient with alcohol dependence (AD). The upper panel shows the location of 16 polymorphisms in *SLC6A3* gene and lower panel shows the output of Haploview version 4.1. D’ (left) and r^2^ value (right) were shown within each square represents a pairwise linkage disequilibrium relationship between two polymorphisms. The definitions of the abbreviations: Ex, exon; In, intron; P, promoter.
(TIF)

S2 Fig The linkage disequilibrium (LD) structure between 16 SNP polymorphisms in *SLC6A3* gene for controls. The upper panel shows the location of 16 polymorphisms in *SLC6A3* gene and lower panel shows the output of Haploview version 4.1. D’ (left) and r^2^ value (right) were shown within each square represents a pairwise linkage disequilibrium relationship between two polymorphisms. The definitions of the abbreviations: Ex, exon; In, intron; P, promoter.
(TIF)

S1 Table Genotype distributions of the polymorphisms in the *SLC6A3* (*DAT*) gene between patients with early-onset alcohol dependence (EOAD) and controls in a Han Chinese population. (DOCX)

S2 Table Genotype distributions and allelic frequencies of the polymorphisms in the *SLC6A3* (*DAT*) gene between male patients with alcohol dependence and controls in a Han Chinese population. (DOCX)
