# Supplementary figures and images for: The SLC6A3 gene possibly affects susceptibility to late-onset alcohol dependence but not specific personality traits in a Han Chinese population
Source: PLoS One. 2017 Feb 9;12(2):e0171170. doi: 10.1371/journal.pone.0171170 (PMC5300170; doi:10.1371/journal.pone.0171170)

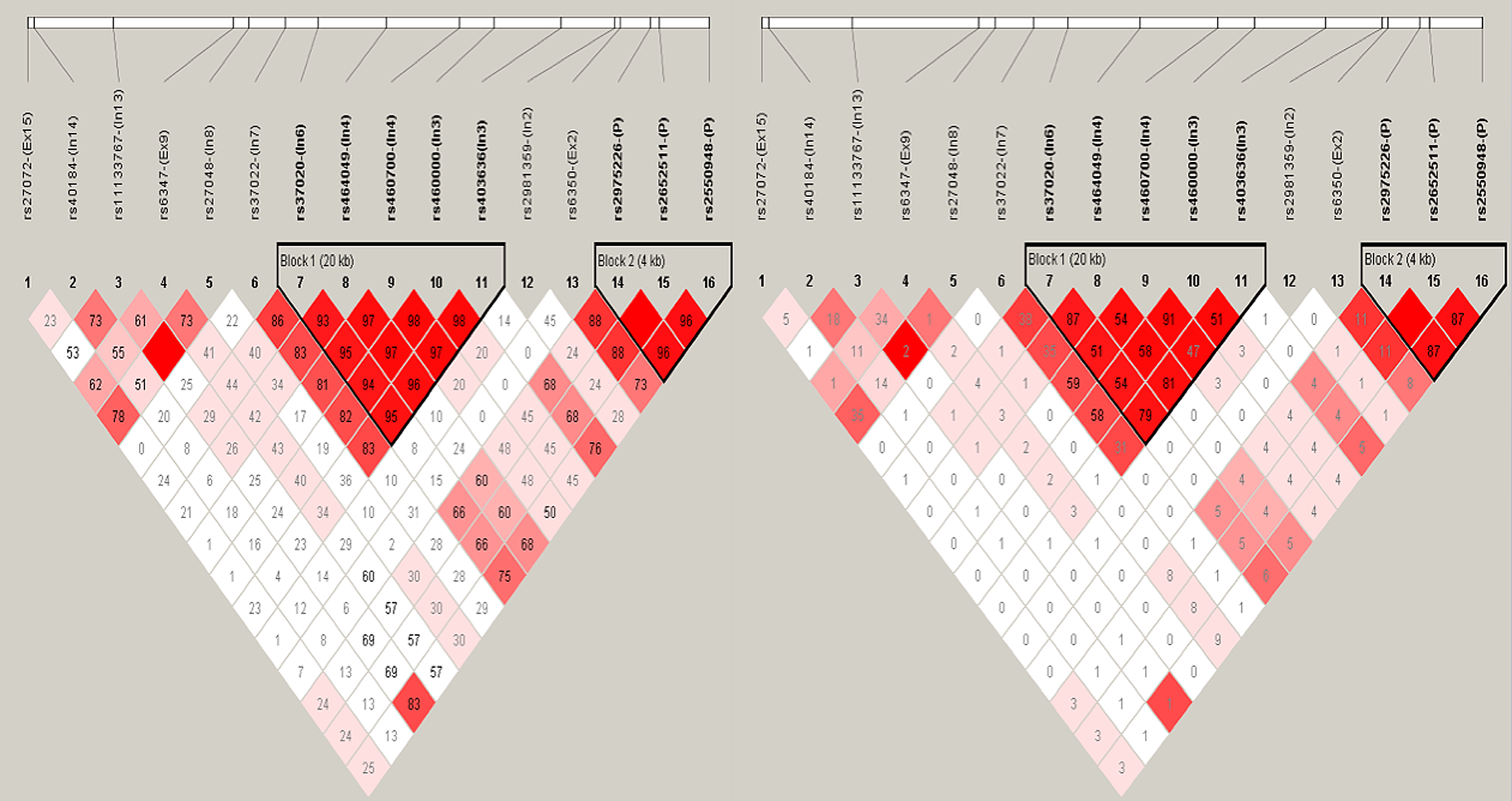

Supplement: S1 Fig — The upper panel shows the location of 16 polymorphisms in SLC6A3 gene and lower panel shows the output of Haploview version 4.1. D’ (left) and r2 value (right) were shown within each square represents a pairwise linkage disequilibrium relationship between two polymorphisms. The definitions of the abbreviations: Ex, exon; In, intron; P, promoter. (TIF) [file pone.0171170.s001.tif]

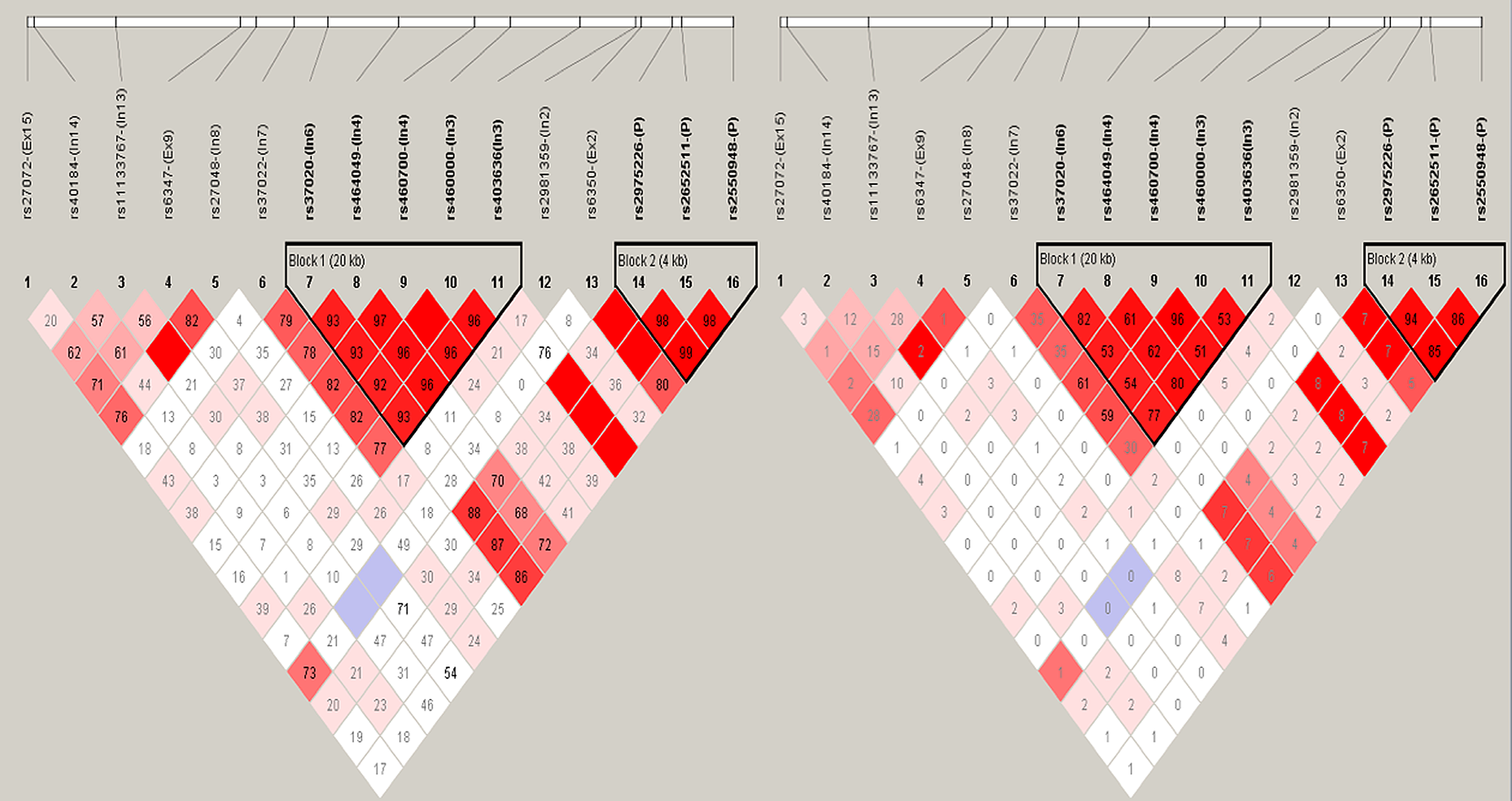

Supplement: S2 Fig — The upper panel shows the location of 16 polymorphisms in SLC6A3 gene and lower panel shows the output of Haploview version 4.1. D’ (left) and r2 value (right) were shown within each square represents a pairwise linkage disequilibrium relationship between two polymorphisms. The definitions of the abbreviations: Ex, exon; In, intron; P, promoter. (TIF) [file pone.0171170.s002.tif]
