# Supplementary material for: The SLC6A3 gene possibly affects susceptibility to late-onset alcohol dependence but not specific personality traits in a Han Chinese population
Source: PLoS One. 2017 Feb 9;12(2):e0171170. doi: 10.1371/journal.pone.0171170 (PMC5300170; doi:10.1371/journal.pone.0171170)
Supplement: S1 Table — (DOCX) [file pone.0171170.s003.docx]

**Supplementary Table 1** Genotype distributions of the polymorphisms in the *SLC6A3 (DAT)* gene between patients with early-onset alcohol dependence (EOAD) and controls in a Han Chinese population

| **Variant** | **Position reference**  **dSNP** | **Allele ^a^** | | **Genotype (%)** | | | | | | | |
| --- | --- | --- | --- | --- | --- | --- | --- | --- | --- | --- | --- |
|  |  | **1** | **2** | **Control (N = 523)** | | |  | **EOAD (N = 229)** | | | ***p*** |
|  |  |  |  | **1/1** | **1/2** | **2/2** |  | **1/1** | **1/2** | **2/2** |  |
| **rs2550948** | **1503444(P)** | ***A*** | ***G*** | 10 (1.9) | 116 (22.2) | 397 (75.9) |  | 3 (1.3) | 63 (27.5) | 163 (71.2) | 0.262^b^ |
| **rs2652511** | **1499389(P)** | ***C*** | ***T*** | 12 (2.3) | 126 (24.1) | 385 (73.6) |  | 3 (1.3) | 66 (28.8) | 160 (69.9) | 0.280^b^ |
| **rs2975226** | **1498616(P)** | ***A*** | ***T*** | 13 (2.5) | 127 (24.3) | 383 (73.2) |  | 3 (1.3) | 66 (28.8) | 160 (69.9) | 0.287^b^ |
| **rs6350** | **1496199(E2)** | ***A*** | ***G*** | 0 (0) | 13 (2.5) | 510 (97.5) |  | 0 (0.0) | 9 (3.9) | 220 (96.1) | 0.346^b^ |
| **rs2981359** | **1495732(In2)** | ***G*** | ***C*** | 95 (18.2) | 263 (50.3) | 165 (31.5) |  | 43 (18.8) | 111 (48.5) | 75 (32.8) | 0.900 |
| **rs403636** | **1491354(In3)** | ***A*** | ***C*** | 53 (10.1) | 239 (45.7) | 231 (44.2) |  | 32 (14.0) | 94 (41.0) | 103 (45.0) | 0.237 |
| **rs460000** | **1485825(In3)** | ***G*** | ***T*** | 104 (19.9) | 274 (52.4) | 145 (27.7) |  | 60 (26.2) | 103 (45.0) | 66 (28.8) | 0.094 |
| **rs460700** | **1482969(In4)** | ***T*** | ***C*** | 111 (21.2) | 270 (51.6) | 142 (27.2) |  | 60 (26.2) | 105 (45.9) | 64 (27.9) | 0.242 |
| **rs464049** | **1476905(In4)** | ***A*** | ***G*** | 69 (13.2) | 243 (46.5) | 211 (40.3) |  | 40 (17.5) | 93 (40.6) | 96 (41.9) | 0.190 |
| **rs37020** | **1471374(In6)** | ***A*** | ***C*** | 61 (11.7) | 248 (47.4) | 214 (40.9) |  | 34 (14.8) | 99 (43.2) | 96 (41.9) | 0.383 |
| **rs37022** | **1468629(In7)** | ***T*** | ***A*** | 137 (26.2) | 253 (48.4) | 133 (25.4) |  | 52 (22.7) | 115 (50.2) | 62 (27.1) | 0.592 |
| **rs27048** | **1465645(In8)** | ***T*** | ***C*** | 12 (2.3) | 140 (26.8) | 371 (70.9) |  | 3 (1.3) | 68 (29.7) | 158 (69.0) | 0.512^b^ |
| **rs6347** | **1464412(E9)** | ***G*** | ***A*** | 4 (0.8) | 122 (23.3) | 397 (75.9) |  | 5 (2.2) | 38 (16.6) | 186 (81.2) | 0.032^b^ |
| **rs11133767** | **1454580(In13)** | ***T*** | ***C*** | 14 (2.7) | 89 (17.0) | 420 (80.3) |  | 4 (1.7) | 35 (15.3) | 190 (83.0) | 0.592^b^ |
| **rs40184** | **1448077(In14)** | ***T*** | ***C*** | 30 (5.7) | 207 (39.6) | 286 (54.7) |  | 14 (6.1) | 99 (43.2) | 116 (50.7) | 0.593 |
| **rs27072** | **1447522(E15)** | ***T*** | ***C*** | 45 (8.6) | 197 (37.7) | 281 (53.7) |  | 11 (4.8) | 88 (38.4) | 130 (56.8) | 0.184 |

MAF, minor allele frequency; P, promoter; E, exon; In, intron.

^a^ Allele 1 is the minor allele, and only alleles with frequency higher than 1 % are shown.

^b^ Statistical analysis was performed by Fisher’s exact test.
