# Supplementary material for: The SLC6A3 gene possibly affects susceptibility to late-onset alcohol dependence but not specific personality traits in a Han Chinese population
Source: PLoS One. 2017 Feb 9;12(2):e0171170. doi: 10.1371/journal.pone.0171170 (PMC5300170; doi:10.1371/journal.pone.0171170)
Supplement: S2 Table — (DOCX) [file pone.0171170.s004.docx]

**Supplementary Table 2** Genotype distributions and allelic frequencies of the polymorphisms in the *SLC6A3 (DAT)* gene between male patients with alcohol dependence and controls in a Han Chinese population

| **Variant** | **Position reference dSNP** | **Allele ^a^** | | **MAF ^a^ (%)** | | ***p* ^b^** | **Genotype (%)** | | | | | | | | | | | | ***p* ^b^** |
| --- | --- | --- | --- | --- | --- | --- | --- | --- | --- | --- | --- | --- | --- | --- | --- | --- | --- | --- | --- |
|  |  | **1** | **2** | **Control** | **AD** |  | **Control (N = 362)** | | | |  | **AD (N = 562)** | | | ***p* ^b^** | **LOAD (N = 362)** | | |  |
|  |  |  |  |  |  |  | **1/1** | | **1/2** | **2/2** |  | **1/1** | **1/2** | **2/2** |  | **1/1** | **1/2** | **2/2** |  |
| **rs2550948** | **1503444(P)** | ***A*** | ***G*** | 13.54 | 16.28 | 0.109 | 6 (1.7) | 86 (23.8) | | 270 (74.6) |  | 12 (2.1) | 159 (28.3) | 391 (69.6) | 0.254 | 9 (2.5) | 101 (27.9) | 252 (69.6) | 0.298 |
| **rs2652511** | **1499389(P)** | ***C*** | ***T*** | 15.06 | 17.17 | 0.230 | 8 (2.2) | 93 (25.7) | | 261 (72.1) |  | 12 (2.1) | 169 (30.1) | 381 (67.8) | 0.353 | 9 (2.5) | 108 (29.8) | 245 (67.7) | 0.431 |
| **rs2975226** | **1498616(P)** | ***A*** | ***T*** | 15.33 | 17.17 | 0.298 | 9 (2.5) | 93 (25.7) | | 260 (71.8) |  | 12 (2.1) | 169 (30.1) | 381 (67.8) | 0.346 | 9 (2.5) | 108 (29.8) | 245 (67.7) | 0.457 |
| **rs6350** | **1496199(E2)** | ***A*** | ***G*** | 1.38 | 2.76 | 0.050 | 0 (0) | 10 (2.08) | | 352 (97.2) |  | 0 (0.0) | 31 (5.5) | 531 (94.5) | 0.047^c^ | 0 (0) | 24 (6.6) | 338 (93.4) | 0.021^c^ |
| **rs2981359** | **1495732(In2)** | ***G*** | ***C*** | 42.82 | 43.77 | 0.686 | 66 (18.2) | 178 (49.2) | | 118 (32.6) |  | 109 (19.4) | 274 (48.8) | 179 (31.9) | 0.903 | 73 (20.2) | 174 (48.1) | 115 (31.8) | 0.804 |
| **rs403636** | **1491354(In3)** | ***A*** | ***C*** | 33.01 | 33.01 | 0.999 | 36 (9.9) | 167 (46.1) | | 159 (43.9) |  | 62 (11.0) | 247 (44.0) | 253 (45.0) | 0.764 | 34 (9.4) | 162 (44.8) | 166 (45.9) | 0.868 |
| **rs460000** | **1485825(In3)** | ***G*** | ***T*** | 47.10 | 49.02 | 0.420 | 68 (18.8) | 205 (56.6) | | 89 (24.6) |  | 131 (23.3) | 289 (51.4) | 142 (25.3) | 0.197 | 78 (21.5) | 193 (53.3) | 91 (25.1) | 0.586 |
| **rs460700** | **1482969(In4)** | ***T*** | ***C*** | 47.65 | 49.73 | 0.382 | 70 (19.3) | 205 (56.6) | | 87 (24.0) |  | 136 (24.2) | 287 (51.1) | 139 (24.7) | 0.128 | 86 (23.8) | 190 (52.5) | 86 (23.8) | 0.330 |
| **rs464049** | **1476905(In4)** | ***A*** | ***G*** | 37.02 | 36.21 | 0.725 | 48 (13.3) | 172 (47.5) | | 142 (39.2) |  | 80 (14.2) | 247 (44.0) | 235 (41.8) | 0.569 | 46 (12.7) | 161 (44.5) | 155 (42.8) | 0.614 |
| **rs37020** | **1471374(In6)** | ***A*** | ***C*** | 35.50 | 36.12 | 0.785 | 41 (11.3) | 175 (48.3) | | 146 (40.3) |  | 81 (14.4) | 244 (43.4) | 237 (42.2) | 0.229 | 52 (14.4) | 155 (42.8) | 155 (42.8) | 0.249 |
| **rs37022** | **1468629(In7)** | ***T*** | ***A*** | 50.41 | 46.98 | 0.149 | 92 (25.4) | 181 (50.0) | | 89 (24.6) |  | 132 (23.5) | 264 (47.0) | 166 (29.5) | 0.258 | 90 (24.9) | 159 (43.9) | 113 (31.2) | 0117 |
| **rs27048** | **1465645(In8)** | ***T*** | ***C*** | 15.33 | 16.28 | 0.586 | 8 (2.2) | 95 (26.2) | | 259 (71.5) |  | 8 (1.4) | 167 (29.7) | 387 (68.9) | 0.377 | 5 (1.4) | 105 (29.0) | 252 (69.6) | 0.525 |
| **rs6347** | **1464412(E9)** | ***G*** | ***A*** | 12.70 | 11.15 | 0.248 | 3 (0.8) | 86 (23.8) | | 273 (75.4) |  | 9 (1.6) | 105 (18.7) | 448 (79.7) | 0.122^c^ | 5 (1.4) | 75 (20.7) | 282 (77.9) | 0.527^c^ |
| **rs11133767** | **1454580(In13)** | ***T*** | ***C*** | 9.53 | 10.94 | 0.501 | 6 (1.7) | 57 (15.7) | | 299 (82.6) |  | 11 (2.0) | 96 (17.1) | 455 (81.0) | 0.812 | 7 (1.9) | 64 (17.7) | 291 (80.4) | 0.744 |
| **rs40184** | **1448077(In14)** | ***T*** | ***C*** | 25.41 | 25.09 | 0.875 | 20 (5.5) | 144 (39.8) | | 198 (54.7) |  | 32 (5.7) | 218 (38.8) | 312 (55.5) | 0.955 | 19 (5.2) | 132 (36.5) | 211 (58.3) | 0.619 |
| **rs27072** | **1447522(E15)** | ***T*** | ***C*** | 28.87 | 24.82 | 0.054 | 37 (10.2) | 135 (37.3) | | 190 (52.5) |  | 33 (5.9) | 213 (37.9) | 316 (56.2) | 0.048 | 24 (6.6) | 136 (37.6) | 202 (55.8) | 0.208 |

MAF, minor allele frequency; P, promoter; E, exon; In, intron; AD: Alcohol dependence; LOAD: late-onset alcohol dependence.

^a^ Allele 1 is the minor allele, and only alleles with frequency higher than 1 % are shown.

^b^ Indicated genotype or allelic frequencies in patients with AD or LOAD compared with the control group.

^c^ Statistical analysis was performed by Fisher’s exact test.
